# Conserved asymmetry underpins homodimerization of Dicer-associated double-stranded RNA-binding proteins

**DOI:** 10.1093/nar/gkx928

**Published:** 2017-10-17

**Authors:** Alex Heyam, Claire E. Coupland, Clément Dégut, Ruth A. Haley, Nicola J. Baxter, Leonhard Jakob, Pedro M. Aguiar, Gunter Meister, Michael P. Williamson, Dimitris Lagos, Michael J. Plevin

**Affiliations:** 1 Department of Biology, University of York, York, YO10 5DD, UK; 2 Department of Molecular Biology and Biotechnology, University of Sheffield, Sheffield, S10 2TN, UK; 3 Biochemistry Center Regensburg (BZR), Laboratory for RNA Biology, University of Regensburg, 93053 Regensburg, Germany; 4 Department of Chemistry, University of York, York, YO10 5DD, UK; 5 Centre for Immunology and Infection, Department of Biology and Hull York Medical School, University of York, Wentworth Way, York, YO10 5DD, UK

## Abstract

Double-stranded RNA-binding domains (dsRBDs) are commonly found in modular proteins that interact with RNA. Two varieties of dsRBD exist: canonical Type A dsRBDs interact with dsRNA, while non-canonical Type B dsRBDs lack RNA-binding residues and instead interact with other proteins. In higher eukaryotes, the microRNA biogenesis enzyme Dicer forms a 1:1 association with a dsRNA-binding protein (dsRBP). Human Dicer associates with HIV TAR RNA-binding protein (TRBP) or protein activator of PKR (PACT), while Drosophila Dicer-1 associates with Loquacious (Loqs). In each case, the interaction involves a region of the protein that contains a Type B dsRBD. All three dsRBPs are reported to homodimerize, with the Dicer-binding region implicated in self-association. We report that these dsRBD homodimers display structural asymmetry and that this unusual self-association mechanism is conserved from flies to humans. We show that the core dsRBD is sufficient for homodimerization and that mutation of a conserved leucine residue abolishes self-association. We attribute differences in the self-association properties of Loqs, TRBP and PACT to divergence of the composition of the homodimerization interface. Modifications that make TRBP more like PACT enhance self-association. These data are examined in the context of miRNA biogenesis and the protein/protein interaction properties of Type B dsRBDs.

## INTRODUCTION

Double-stranded (ds) RNA-binding domains (dsRBDs; also called dsRNA-binding motifs or dsRBMs) are found in all domains of life, and contribute to diverse biological processes ranging from splicing to antiviral responses (1,2). All dsRBDs adopt a common α-β-β-β-α fold, but they can be divided into two distinct classes: those that bind dsRNA and those that do not (3). Type-A dsRBDs bind dsRNA via three conserved regions. This interaction rarely displays any specificity for RNA sequence and is instead dependent on dsRNA-specific groove structures and the 2′-OH of the ribose sugar (1). Type B dsRBDs lack residue conservation in dsRNA recognition regions, including two critical lysine residues in dsRNA recognition Region 3, and consequently cannot bind dsRNA (3). Instead, Type B dsRBDs have evolved to mediate protein–protein interactions. While there have been many structural studies of Type A dsRBDs, only recently has structural information about protein–protein interactions mediated by Type B domains become available.

DsRBDs are commonly found in proteins that contain other RNA-binding or RNA-processing domains, such as the RNase III domains in Dicer and Drosha, the kinase domain in interferon-induced, dsRNA-activated protein kinase (PKR), or the A-to-I deaminase domain in dsRNA-specific adenosine deaminase (ADAR). In eukaryotes, dsRNA-binding proteins can contain multiple dsRBDs, either of a single type or a mixture of Type A and Type B. Sequence similarity between dsRBDs in the same protein can vary considerably. Studies that have characterized the properties of individual Type A domains from multi-dsRBD proteins have revealed that they typically show different affinities for dsRNA (4–8). These data suggest that multiple dsRBDs offer a convenient way to tune protein affinity to dsRNA and, through the protein binding properties of Type B dsRBDs, to interact with other dsRNA binding proteins.

In microRNA (miRNA) biogenesis, a protein complex containing a Dicer enzyme processes precursor miRNAs (pre-miRNAs). Eukaryotic Dicers typically associate with one or more dsRBD-containing protein. In humans, Dicer associates with one of two homologous proteins: protein activator of PKR (PACT) or TAR-RNA binding protein (TRBP) (9–11). In *Drosophila*, pre-miRNAs are processed by Dicer-1, which associates with Loquacious (Loqs), a homologue of both TRBP and PACT (12).

TRBP, PACT and Loqs contain three dsRBDs, two Type A and one Type B. All three proteins interact with their respective Dicers via the Type B dsRBD, which shows high sequence conservation across the three proteins (Supplementary Figure S1) (3,13–15). PACT and TRBP are also implicated in the regulation of PKR, with dsRBD 3 (D3) being responsible for inhibition in the case of TRBP, and conditional activation in PACT (16–18). Each protein has also been reported to homodimerize, an interaction mediated by a region in the C-terminus that contains the Type B dsRBD (6,19,20). The structural biology of homodimerization of non-canonical dsRBDs has only recently been interrogated. Homodimer crystal structures of dsRBDs from HYL1 (7), Staufen (21) and Loqs (20) have all recently been reported with each structure proposing a different homodimerization interface. Knowledge of the dsRBD interfaces and mechanisms that promote homomeric interactions in these proteins is critical as these dsRBDs also mediate heteromeric interactions with functional binding partners, such as the interaction of TRBP, PACT and Loqs with Dicer (22) and the interaction of Staufen with Miranda (23).

Here, we show that homodimerzation of full-length PACT is mediated exclusively via the Type B dsRBD (PACT-D3) and that this property is not dependent on dsRNA. We examine the homodimerization domain and reveal significant structural asymmetry at the homodimerization interface. Nuclear magnetic resonance (NMR) spectroscopic analysis and mutagenesis reveal that PACT-D3, TRBP-D3 and Loqs-D3 all homodimerize using the same surface and molecular mechanism. The conserved asymmetry in the homodimerization of PACT, Loqs and TRBP is an inherent property of these domains. We show that asymmetry results from the formation of an inter-molecular parallel β-sheet, which is stabilized by an inter-strand hydrogen bonding network that would not be possible in a symmetric parallel dimer. We show that TRBP-D3 dimerizes more weakly than PACT-D3 but that a two amino acid substitution in TRBP-D3, which makes the homodimerization interface more PACT-like, stabilizes asymmetric homodimerization. To our knowledge, the asymmetric β-sheet described here is a novel symmetry-breaking motif in homomeric protein oligomerization. The same surface forms the binding site for Dicer (22), suggesting a link between asymmetric homodimerization in TRBP, PACT and Loqs, and their role in miRNA biogenesis.

## MATERIALS AND METHODS

### Plasmid construction

Codon-optimized sequences of PACT and TRBP were ordered from GeneArt, and regions corresponding to PACT residues 239–313 (PACT-D3) and 208–313 (PACT-Ext-D3), and TRBP residues 258–366 (TRBP-Ext-D3) were cloned into a vector derived from pET-28a (24) using an In-Fusion cloning strategy (Clontech). This vector is based on the pET vector series, and results in attachment of an N-terminal hexa-histidine tag, maltose binding protein (MBP) and HRV 3C protease cleavage site. Loqs-D3 (residues 392–463) was cloned into the pGEX-4T-1 plasmid as described previously (20). Mutations were introduced using QuikChange Lightning mutagenesis kits (Agilent).

### Protein expression and purification

Proteins were expressed in *Escherichia coli* BL21 (DE3) grown in M9 minimal media containing ^15^N ammonium chloride (and ^13^C glucose for samples used for 3D NMR experiments). Cultures were grown to an OD_600_ of 0.6–0.8 at 37°C, then induced with 1 mM isopropyl-beta-D-thiogalactopyranoside and further incubated at 20°C for 16 h. For PACT-D3, PACT-Ext-D3 and TRBP-Ext-D3, cells were lysed by sonication or by continuous flow French press. His-MBP-tagged protein was purified using HisTrap FF columns (GE Healthcare). The fusion proteins were incubated overnight with His-tagged 3C protease, before being passed over a HisTrap FF column to remove the tag and protease. The domain of interest was then further purified using a Superdex S75 16/60 size exclusion column (GE Healthcare), equilibrated with 20 mM MES pH 6.5, 50 mM NaCl, before a final dialysis against 20 mM MES pH 6.5, 50 mM NaCl, and 5–10 mM TCEP. For Loqs-D3, purification was as above, except GSTrap columns (GE Healthcare) were used instead of HisTrap FF, tobacco etch virus protease was used instead of HRV 3C protease, and all purification steps except size exclusion chromatography were performed at 4°C. Protein concentration was determined by absorbance measurements at 280 nm.

### SEC-MALLS

Except where otherwise noted, a Superdex S75 10/30 analytical column (GE Healthcare) was equilibrated with 20 mM MES pH 6.5, 200 mM NaCl, 1 mM dithiothreitol at a flow rate of 0.5 ml/minute. A total of 100 μl of protein sample (at 2–5 mg/ml) was injected, and refractive index and light scattering profiles were recorded inline using Wyatt rEX Optilab and Wyatt Dawn HELEOS-II instruments. Data were analysed using ASTRA software version 5.3.4.14 (Wyatt Instruments), using a Zimm model. Light scattering detectors were normalized on a sample of BSA, and dn/dc chosen in the range 0.164–0.180 to give the correct mass for BSA. All SEC MALLS experiments of TRBP and PACT constructs were conducted at least twice.

### NMR data

NMR samples were prepared by dialysis into 20 mM MES pH 6.5, 50 mM NaCl, 5–10 mM TCEP followed by the addition of 10% D_2_O and 50 μM 4,4-dimethyl-4-silapentane-1-sulfonic acid (DSS). The 2D (^1^H, ^15^N) HSQC and EXSY spectra, and 3D experiments for assignment of PACT-D3 L273R, were recorded using a Bruker Avance II 700 MHz spectrometer with a triple-resonance room temperature probe. Spectra for backbone assignment of wild-type (WT) PACT-D3 were recorded on a Bruker 600 MHz Avance II+ spectrometer with triple-resonance cryoprobe, while spectra for side-chain assignment was collected on a Bruker 800 MHz Avance III HD spectrometer with triple-resonance cryoprobe. The ^13^C filter-edit NOESY experiment was recorded on a 50:50 mixture of [^13^C,^15^N]- and [^15^N]-labelled WT PACT-D3 using a Bruker 700 MHz Avance III HD spectrometer with quadruple-resonance cryoprobe. The high pressure 2D (^1^H, ^15^N) HSQC NMR experiments were recorded using a Bruker 800 MHz Avance I spectrometer, equipped with a triple-resonance room temperature probe. The sample was inserted into a ceramic tube (rated to 2.5 kbar) and pressurized with paraffin oil (Sigma) using a high-pressure syringe pump (Daedalus Innovations LLC, PA).

### NMR data analysis

Spectra were processed with either TopSpin (Bruker) or NMRPipe (25). Assignment of PACT-D3 backbone and sidechain resonances, and peak picking of EXSY spectra, was performed with CCPNMR Analysis V2 (26). A ‘compound’ chemical shift difference was calculated as $\sqrt {\delta _H^2 + {{({\delta _N}/6.5)}^2})}$. EXSY data were analysed using a ratio of auto- and crosspeak intensities (see Supplementary Data) (27).

### Modelling and structural analysis

The structural model of PACT-D3 was generated using the I-TASSER server (28). Analysis of the surface buried upon dimerization of Loqs-D3 was performed using the POPS server, and averaged between the two dimer subunits (29).

## RESULTS

### The mechanism of homodimerization of PACT-D3 and Loqs-D3 is equivalent

Modular, multidomain proteins can often be dissected into isolated functional units. TRBP, Loqs and PACT have been reported to have both dsRNA and protein–protein binding properties, with these activities principally mapping to dsRBDs 1 and 2, and dsRBD 3, respectively. We produced a series of constructs of PACT to explore the structure/function profile of the protein and determined their oligomeric state and pre-miRNA binding properties. We conducted size exclusion chromatography with inline multi-angle laser light scattering (SEC-MALLS), which showed that full length (FL) PACT is a homodimer in solution (Supplementary Figure S2A). Homodimerization is dependent on dsRBD 3, as a construct lacking this domain (PACT-D12) eluted with a mass consistent with a monomer (Supplementary Figure S2B). Furthermore, an analysis of the pre-miRNA binding properties of PACT using electrophoretic mobility shift assays (EMSA) revealed that dsRNA binding mapped exclusively to dsRBDs 1 and 2, consistent with previous studies of PACT (30,31). FL PACT and PACT-D12, but not PACT-D3, were able to bind pre-miR-155 (Supplementary Figure S2C).

**Figure 1.**
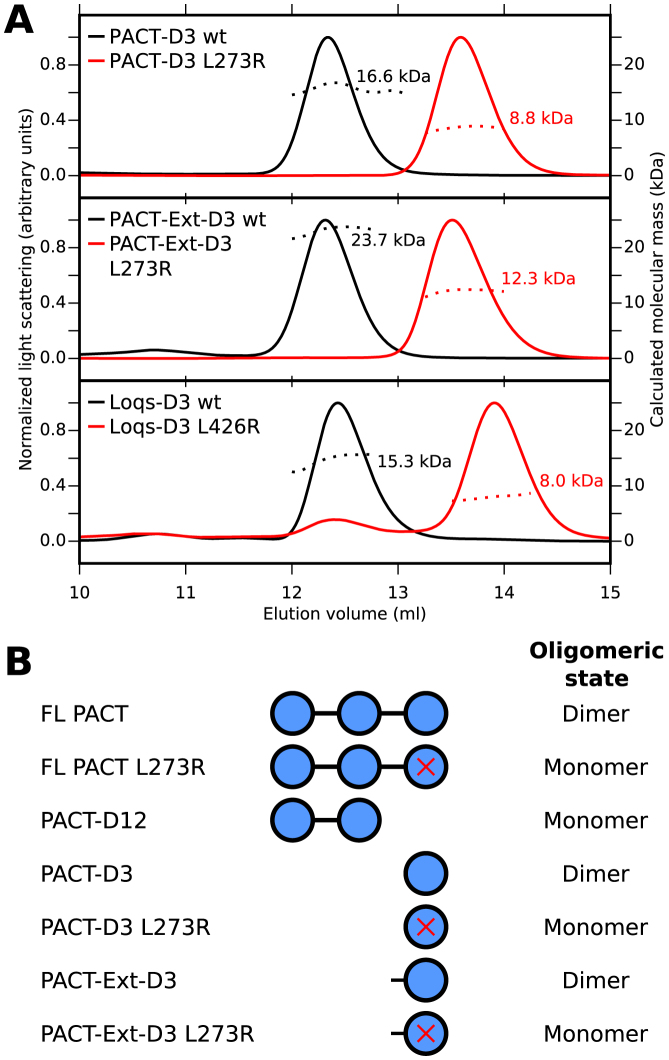
Homodimerization of PACT is mediated exclusively via domain 3. (**A**) SEC-MALLS results for PACT and Loqs domain 3 constructs. WT domains are shown in black, mutants are shown in red. The calculated molecular mass at the peak centre is displayed next to each peak. (**B**) Summary of oligomeric states of single- and multi-domain PACT constructs (see also Supplementary Figure S2).

The homodimer structure of *Drosophila* Staufen-D5 revealed that a 20 residue region N-terminal to the dsRBD was required for homodimerization (21). TRBP and PACT have a highly conserved 15-residue region N-terminal to dsRBD 3 (Supplementary Figure S1). We examined the contribution of this region to the dimerization properties of PACT. Both the core dsRBD (PACT-D3) and a construct containing the conserved N-terminal extension (PACT-Ext-D3) eluted with masses consistent with homodimers (Figure 1A). DsRBD 3 in Loqs is also preceded by an N-terminal extension, which shows some sequence conservation to PACT and TRBP (Supplementary Figure S1). However, like PACT-D3, Loqs-D3 eluted with a molecular weight consistent with a homodimer (Figure 1A). These data are consistent with a homodimer observed in the recent crystal structure of the core dsRBD of domain 3 of Loqs (20).

Homodimerization of a construct containing Loqs-D3 could be disrupted by a single point mutation (L426R) on the homodimer interface (20). The equivalent mutation in PACT (L273R) abolished homodimerization in both PACT-D3 and PACT-Ext-D3, showing that the presence of the N-terminal region does not compensate for mutations in the dsRBD core (Figure 1A). The mutation Y305A also disrupted homodimerization of PACT-D3, consistent with the location of this conserved tyrosine at the homodimer interface, but a number of other mutations on the same surface did not (data not shown). Taken together, these solution studies show that the core dsRBD fold of domain 3 in both Loqs and PACT is necessary and sufficient for homodimerization (Figure 1B), and suggest that PACT-D3 and Loqs-D3 share a common mechanism of homodimerization.

### Two different states of PACT-D3 are present in solution

**Figure 2.**
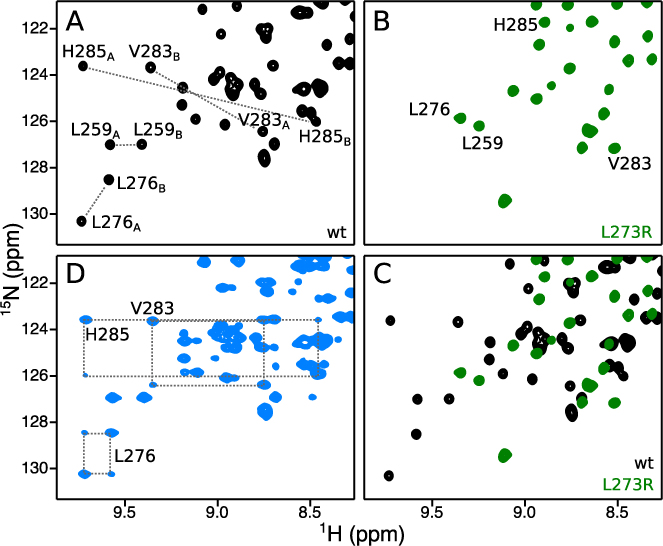
PACT-D3 has two distinct states in solution. (**A**–**C**) (^1^H, ^15^N)-HSQC spectra of (A) WT PACT-D3, (B) PACT-D3 L273R, or (C) both, with selected assignments. (**D**) EXSY spectrum of WT PACT-D3. Selected sets of auto and exchange peaks are linked with dotted lines.

NMR spectroscopy was used to further characterize homodimerization of PACT-D3. Following resonance assignment (see Supplementary Methods), we observed that NMR spectra of WT PACT-D3 contained two signals per site (Figure 2A and Supplementary Figure S3A), indicating that at least two distinct states are present in solution (arbitrarily named A and B). By contrast, spectra of PACT-D3 L273R contained only one signal per site (Figure 2B and Supplementary Figure S3B). Approximately twice the expected number of cross peaks were also observed in NMR spectra of WT PACT-Ext-D3 and WT Loqs-D3, while the monomeric mutant forms of both constructs gave closer to the expected number of signals (Supplementary Figure S4).

Analysis of backbone chemical shifts in WT PACT-D3 using TALOS-N (32) showed no significant differences in secondary structure between states A and B (Supplementary Figure S3C). Moreover, the relative intensities of peaks in a 2D (^1^H,^15^N) correlation spectra were approximately equal, indicating that the two states are equally populated. This observation was true for a range of conditions, including high pressure (2.5 kbar), low concentration of denaturant (2.5 M urea) and across a range of temperatures (Supplementary Figure S3D).

We used 2D heteronuclear NMR exchange spectroscopy (EXSY) to determine whether the two states of WT PACT-D3 are in exchange with one another. With a mixing time of 0.5 s, exchange cross peaks were observed for almost all residues (Figure 2D and Supplementary Figure S5A), suggesting a global exchange process. A series of 2D EXSY spectra was used to calculate a global exchange rate of 0.71 s^−1^, with a 95% confidence interval of (0.65 s^−1^, 0.86 s^−1^), assuming equal populations of the two states (Supplementary Figure S5B).

### PACT-D3 homodimers are asymmetric

We ruled out a number of potential explanations for the two states of WT PACT-D3 that were observed in NMR spectra (see Supplementary Table S1), which left two plausible explanations: the two sets of signals could originate from two different homodimeric forms of WT PACT-D3, both of which are symmetric; or WT PACT-D3 could form a single asymmetric homodimer, with each half of the dimer giving rise to a separate set of signals in NMR spectra.

**Figure 3.**
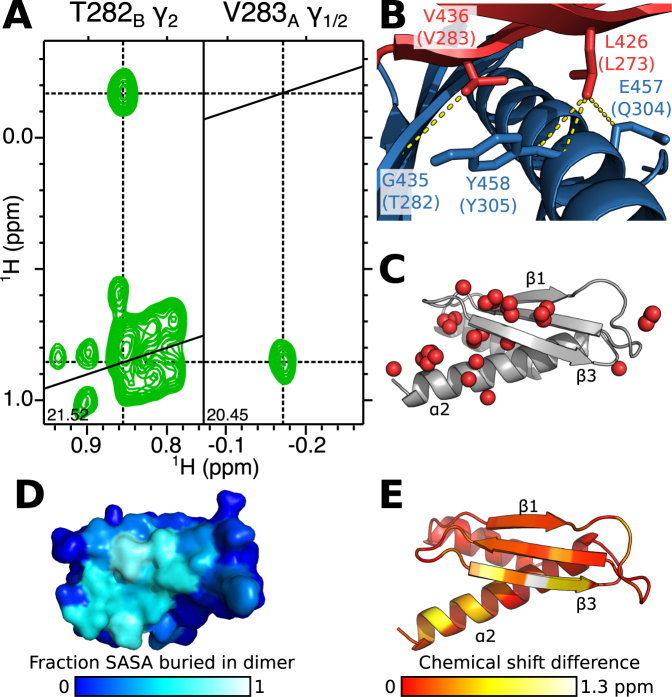
Homodimerization of PACT-D3 involves the same interface as Loqs-D3. (**A**) 2D (^1^H,^1^H) planes from the 3D ^13^C-filtered NOESY HSQC spectrum of PACT-D3 showing an intermolecular NOE between one of the γ methyl groups from V283_A_ and the γ_2_ methyl group of T282_B_. (**B**) In the 3D structure of Loqs-D3, residues equivalent to PACT L273 and V283 in state A (red) form intermolecular contacts with residues equivalent to T282, Q304 and Y305 in state B (blue). (**C**) Interface atoms in PACT-D3 identified from the 3D ^13^C-filtered NOESY HSQC spectrum (red spheres) displayed on a 3D model of PACT-D3. (**D**) Interface of Loqs-D3 displayed on a single subunit as the proportion of solvent accessible surface area (SASA) which is buried upon homodimerization. (**E**) Compound chemical shift differences of the backbone amides between the two states of WT PACT-D3, mapped onto a structural model. Red indicates residues with low chemical shift changes, yellow and white indicate larger shifts. Panels C and E show a model of PACT-D3 generated using I-TASSER.

To distinguish conclusively between these possibilities, we prepared a sample containing equimolar amounts of natural abundance, and [^13^C,^15^N]-labelled PACT-D3, and recorded a ^13^C-filtered NOESY NMR experiment (Supplementary Figure S6). This NMR experiment detects NOEs of intermolecular origin thereby allowing direct identification of sites at the homodimer interface. Moreover, this experiment can distinguish symmetric versus asymmetric homodimerization: in a symmetric homodimer, intermolecular NOEs would only be observed between nuclei of the same state (i.e. only A to A or B to B), while an asymmetric homodimer would have NOEs linking the two different states (i.e. only A to B or B to A). ^1^H-^1^H NOE cross peaks were observed between one of the γ-methyl groups of V283 in state A, and the γ_2_-methyl group of T282 in state B (Figure 3A and Supplementary Figure S6C) and between one of the δ-methyl groups of L273 in state A and the α- and β-protons of Q304 and Y305 in state B (Supplementary Figure S6D). These NOE correlations would only be possible if WT PACT-D3 forms an asymmetric homodimer.

The 3D structure of Loqs-D3 was initially described as forming a symmetric homodimer (20), but in light of the NMR-based data presented here, re-inspection of the structure revealed that the protomers in fact associate to form an asymmetric homodimer (Supplementary Figure S7). The equivalent residues of L273, V283, Q304 and Y305 in Loqs are located at this asymmetric interface (Figure 3B). Partial assignment of a number of ^13^C-filtered NOESY signals originating from the β-sheet and C-terminal α-helix indicate that they are also located on the homodimer interface (Figure 3C). These data further support the conclusion that PACT-D3 homodimerizes via the same interface that was identified in the Loqs-D3 homodimer (Figure 3D) and that this association forms an asymmetric homodimer.

### Asymmetry is localized to the homodimer interface

The degree of asymmetry of PACT-D3 homodimers at a given location can be qualitatively described by the chemical shift difference between equivalent nuclei in the two states. Plotting these values on a structural model of PACT-D3 revealed that the greatest differences occur on strand β3 and the C-terminal half of helix α2. Smaller differences are visible on the other β-strands (Figure 3E). The largest chemical shift differences occur at residues V283 and H285. Overall, the most significant chemical shift differences occur predominantly at the homodimer interface, and, given the similar TALOS-N profiles of the two states, these chemical shift differences are unlikely to be caused by conformational changes in other parts of the protein.

**Figure 4.**
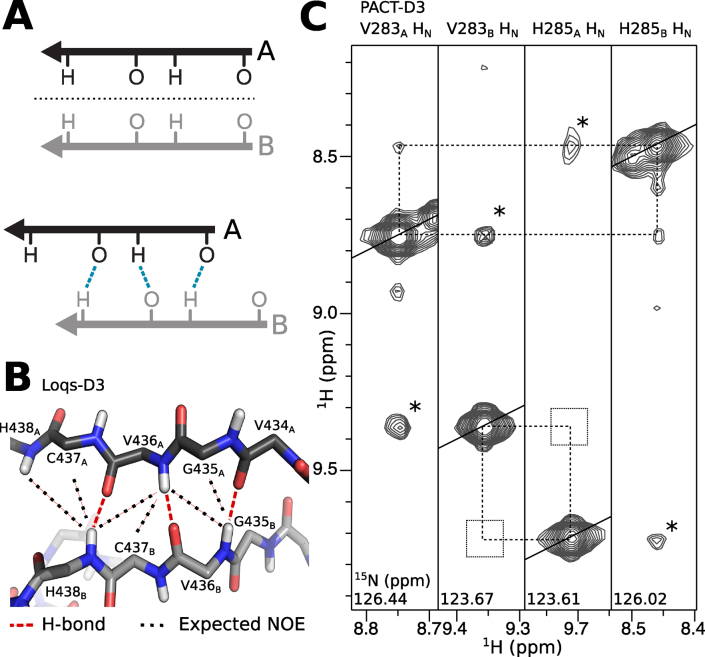
Asymmetric homodimerization forms an inter-subunit parallel β-sheet. (**A**) Upper: symmetric homodimer interfaces cannot involve parallel β-strands because the symmetry axis (dashed line) prevents alignment of hydrogen bond donors and acceptors. Lower: offsetting the β-strands allows the formation of hydrogen bonds, but breaks the symmetry. (**B**) Close-up of the Loqs-D3 homodimer interface surrounding V436. Hydrogen bonds are shown as dashed red lines, while predicted NOEs are shown as black dotted lines. (**C**) Planes from a 3D ^15^N-NOESY-HSQC of PACT-D3, showing that NOEs are detected between the backbone amides of V283_A_ and H285_B_ (peaks marked by dotted lines) but not V283_B_ and H285_A_ (empty squares). Cross peaks marked * are due to chemical exchange, and do not necessarily imply proximity. The contour levels are identical in all strips.

In Loqs-D3, V436 and H438 lie at the homodimer interface, where they form part of an inter-subunit parallel β-sheet. Analysis of the 3D structure of Loqs-D3 shows that the two β-strands at the interface are shifted with respect to each other. This configuration breaks the symmetry but allows the formation of intermolecular inter-strand hydrogen bonds: V436_A_ forms hydrogen bonds to V436_B_ and H438_B_, while V436_B_ is bonded to V434_A_ and V436_A_ (Figure 4A and B). The equivalent residues of V436 and H438 in PACT are V283 and H285. To determine whether PACT-D3 homodimerizes via a similar mechanism, we recorded 3D ^15^N-NOESY-HSQC spectra of WT PACT-D3. We found a network of NOEs that is consistent with a parallel β-sheet interface and a similar shift in strand register: the amide protons of V283_A_ and H285_B_ in PACT are in close proximity, but this is not the case for V283_B_ and H285_A_ (Figure 4C).

### A PACT-like variant of TRBP-D3 forms a stronger homodimer

**Figure 5.**
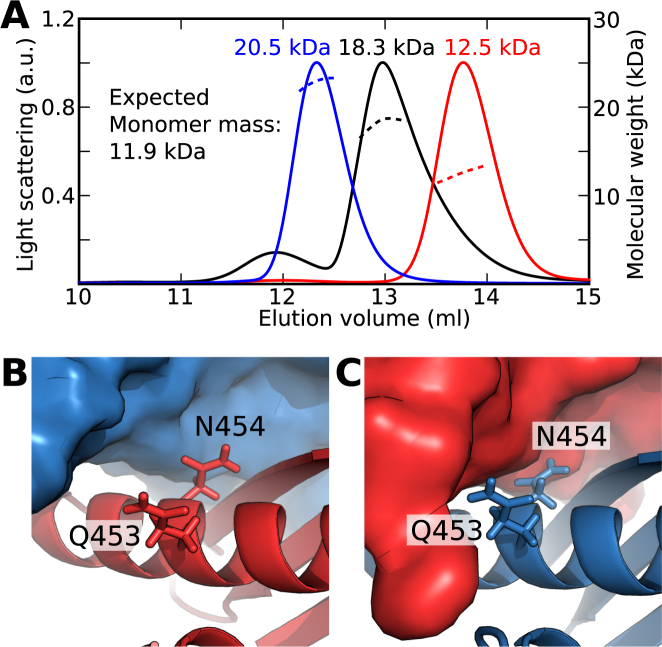
Homodimerization of TRBP-D3 is disfavoured by arginines 353 and 354 at the homodimer interface. (**A**) SEC-MALLS profiles of TRBP-Ext-D3 (black), L326R (red) and R353H, R354N (blue). The single peak of intermediate calculated mass for TRBP-Ext-D3 indicates that the monomer and homodimer forms exchange at a rate faster than approximately 10^−3^ s^−1^. (**B**) Q453 and N454 of Loqs-D3 protomer A (red) are not in direct contact with protomer B (blue). (**C**) By contrast, Q453 and N454 of Loqs-D3 protomer B are in direct contact with protomer A.

The dimerization interface in WT PACT-D3 and WT Loqs-D3 is well conserved in TRBP-D3 (Supplementary Figure S1), and it would therefore be expected that they display similar homodimerization behaviour. However, although full-length TRBP has been shown to homodimerize, a separate study found that TRBP-D3 did not form homodimers (6,19). To further evaluate the homodimerization properties of TRBP, we expressed and purified a construct containing dsRBD 3 and the N-terminal extension (TRBP-Ext-D3). SEC-MALLS analysis of TRBP-Ext-D3 yielded a chromatogram with a single peak with a mass ∼1.5 times that expected for a monomer (Figure 5A). NMR spectra of TRBP-Ext-D3 are characterized by broad line widths, and show considerably less than the expected number of peaks, features which are indicative of exchange broadening (Supplementary Figure S8A). These data indicate that TRBP-Ext-D3 is in monomer/homodimer equilibrium and that it homodimerizes with lower affinity than PACT-D3 and Loqs-D3.

Closer examination of the Loqs-D3 homodimer interface revealed two residues (Q453, N454) that are exposed on one protomer, but form part of the interface on the other (Figure 5B and C). The equivalent residues in PACT-D3 retain similar chemical properties (H300, N301), but in TRBP-D3 they are replaced by arginines (R353, R354). To test whether these differences are responsible for the lower homodimerization affinity of TRBP-Ext-D3, we substituted the two arginine residues with the equivalent residues in PACT-D3. The resulting PACT-like variant of TRBP-Ext-D3 was homodimeric, as assessed by SEC-MALLS (Figure 5A). Moreover, NMR spectra of this R353H,R354N TRBP-Ext-D3 construct showed significantly more than the expected number of peaks (Supplementary Figure S8B). By contrast, the L326R variant of TRBP-Ext-D3 (the equivalent of Loqs-3 L426R mutation) was monomeric and gave rise to a single set of NMR peaks (Figure 5A and Supplementary Figure S8C). These data are consistent with TRBP-Ext-D3 dimerizing via the same asymmetric interface as PACT-D3 and Loqs-D3, with the presence of R353 and R354 residues causing the reduced homodimerization affinity.

## DISCUSSION

Homodimers are the simplest form of protein complex, and account for around 60% of homomeric complexes in the 3D complex database (33). Although many homodimers exhibit some limited local asymmetry, a recent survey found that 90% were globally symmetric (34). Of the remaining structures, 5% exhibited partial asymmetry while only 5% were grossly asymmetric. The heavy bias in favour of symmetry is likely due to a number of factors. For example, symmetric organizations guarantee finite assembly as each subunit has an interface that is satisfied by homomeric association. This prevents formation of higher oligomers such as fibrils (35). In addition, homo-oligomers can also evolve in fewer steps, as each mutation that forms a new favourable interaction is reciprocated by the other subunit, which results in two new interactions overall (36,37). This picture is supported by several computational studies using either idealized interfaces or docking of real protein structures (38,39). It is also possible that structural data of asymmetric homomeric complexes might be under-represented due to technical challenges. Asymmetric homodimerization places each nucleus in two distinct chemical environments, leading to peak doubling in NMR spectra, which confounds the elucidation of the 3D structure via NOE measurements. Asymmetric association of protomers may also disfavour the formation of diffraction-quality protein crystals. These effects would likely reduce the number of 3D structures of asymmetric homodimers deposited in the PDB.

There are myriad ways to break symmetry in a macromolecular assembly. On a molecular level, differences in rotameric states mean that protein subunits are unlikely to ever achieve perfect symmetry. On a more macromolecular level, gross asymmetry can be caused when a symmetric homomer interacts with an odd number of ligands (34). In such cases, asymmetry arises due to a symmetry mismatch between the ligand(s) and homomeric receptor. Other assemblies have been found to exhibit asymmetry based around simple motifs, such as register slips in antiparallel β-strands or coiled coils (40). None of these explanations accounts for homodimerization of PACT, TRBP and Loqs, where the asymmetric association causes a register shift between parallel β-strands at the homodimer interface (Figure 4A). Backbone hydrogen bonding between equivalent parallel β-strands can only occur in an asymmetric homodimer, as symmetric association would cause misalignment of backbone hydrogen bond donors and acceptors (Figure 4A). This asymmetric β-sheet motif of TRBP, PACT and Loqs appears to represent a previously unobserved method of symmetry-breaking in homomeric protein oligomers.

The presence of asymmetry in PACT, TRBP and Loqs homodimers means that, for a given protomer, there are two possible ways the second protomer can associate to form a homodimer. Association occurs via two different, but overlapping, interfaces (Supplementary Figure S7). In this case, the overlap of these interfaces prevents the formation of unbounded fibrils. By way of contrast, the putative dimerization interface reported for dsRBD 2 of HYL1 is asymmetric but without overlap, which would allow the assembly of non-finite fibrils (7).

The homodimerization interface of dsRBD 3 of PACT, TRBP and Loqs is conserved in vertebrates, insects and molluscs (Supplementary Figure S1) and, as such, the asymmetric mechanism of association is likely to have been present in the last common ancestor of these species, at least 580 million years ago (41). In particular, the leucine residue at position 273 in PACT, whose mutation to arginine prevents homodimerization, is conserved in all sequences examined. The conservation at the interface strongly suggests a functional relevance for the asymmetric homodimerization seen in these proteins.

DsRBD 3 of PACT, TRBP and Loqs associates with the helicase insert (Ins) domain of Dicer. Residues that form the Dicer binding interface of TRBP-D3 are well conserved in PACT-D3 and Loqs-D3: TRBP to PACT shows 69% sequence identity; while TRBP to Loqs is 59% (Supplementary Figure S9). It is therefore likely that both Loqs and PACT interact with their respective Dicers in a similar manner to that previously revealed by the 3D structure of the Dicer-TRBP interface (22). In support of this statement, mutation of Dicer prevented interactions with both TRBP and PACT (22). One key difference between the β-sheet surface of PACT, TRBP and Loqs is that TRBP has two positively charged residues (R353, R354) where Loqs and PACT have polar residues (Q453 and N454, or H300 and N301, respectively). Mutating the two arginine residues in TRBP to make them PACT-like increased the stability of the TRBP-D3 homodimer. The 3D structure of the TRBP-Ext-D3/Dicer-Ins complex revealed that R354 of TRBP forms an intermolecular salt bridge with E278 in Dicer (22). A glutamate or aspartate is present at this position in most mammalian Dicers whereas *Drosophila* Dicer1 has no equivalent negative charge in this region. These data suggest that TRBP may have evolved residues that promote interaction with Dicer over homodimerization. Indeed, differences in affinities of homodimerization between TRBP and PACT have been suggested to have implications on how they interact with dsRNA (31). Such differences could potentially allow Dicer to discriminate between dsRNA substrates bound to TRBP and those bound to PACT.

Outside of miRNA processing, knowledge of the structural biology of the protein binding properties of non-canonical Type B dsRBDs has been limited to studies of Staufen from *Drosophila* and human. Homodimer and heterodimer structures of the fifth dsRBD of Staufen (Staufen-D5) have been reported (21,23). There is limited amino acid conservation between D3 of TRBP, PACT and Loqs and Staufen-D5—only the core structural residues that define the dsRBD fold are conserved. However, these domains are all Type B dsRBDs and lack residues related to dsRNA binding.

Staufen-D5 forms a symmetric homodimer, which is mediated by a domain-swap mechanism involving an N-terminal extension to the core dsRBD structure (21). PACT, TRBP and Loqs all have a 15-residue element N-terminal to D3. However, our data conclusively show that this N-terminal extension is not required for homodimerization. The 3D structure of a Staufen-D5/Miranda complex revealed differences between how this domain homo- and heterodimerizes (23). Staufen-D5 interacts with Miranda via the 3-stranded β-sheet of the dsRBD. While this is the same surface involved in TRBP, PACT and Loqs homodimerization, and the interaction between TRBP and Dicer, there is only limited conservation of surface residues between the three Dicer cofactors and Staufen-D5. That said, mutation of isoleucine 982 to alanine in Staufen-D5, the equivalent residue of L273 in PACT, L326 in TRBP and L426 in Loqs, prevented interaction with Miranda (23). Together, these data suggest that unrelated Type B dsRBDs may have evolved different and context-dependent mechanisms for forming protein–protein interactions. However, the β-sheet of dsRBDs forms part of the interface in three of the four complexes of known 3D structure and so it is plausible that this region has properties particularly suited to protein–protein interactions.

Here, we have shown that the Type B dsRBD that mediates interactions between Dicer and its pre-miRNA-binding cofactors forms an asymmetric homodimer and that this asymmetry is conserved from *Drosophila* to humans. Asymmetric association causes a register shift between the parallel β-3 strands, which permits the formation of intermolecular inter-β-strand hydrogen bonds. We identified differences in residue composition of the homodimer interfaces of these Dicer co-factors and showed that mutation of the interface of TRBP to make it more PACT- and Loqs-like enhances homodimerization. These data reveal a rare but conserved example of asymmetric homomeric association, which sheds light on the co-evolution of Dicer and its cofactors.

## AVAILABILITY

Backbone and side-chain resonance assignments for WT PACT-D3 and PACT-D3 L273R have been deposited at BioMagResBank under accession codes 27148 and 27143, respectively.

## Supplementary Material

Supplementary Data
