# Supplementary material for: Conserved asymmetry underpins homodimerization of Dicer-associated double-stranded RNA-binding proteins
Source: Nucleic Acids Res. 2017 Oct 17;45(21):12577–84. doi: 10.1093/nar/gkx928 (PMC5716075; doi:10.1093/nar/gkx928)
Supplement: Supplementary Data [file gkx928_supp.pdf]

Supplementary methods

Electrophoretic mobility shift assays (EMSAs)

A range of sample protein concentrations (0.1-1000 µM) were prepared and incubated with 200 nM Cy5-pre-miR-155 RNA (GE Healthcare Dharmacon) for 1 h at 22 °C. The samples were loaded to non-denaturing native gels (8 % acrylamide, 1 x TBE buffer, 0.1 % APS, TEMED) and run in 0.5 x TBE buffer at 80 V for 90 min at 4 °C. The gels were visualised after 10 min incubation with SYBR® Gold Nucleic Acid Gel Stain (Thermo Fisher Scientific). All EMSAs were repeated at least twice.

Chemical shift assignment of WT PACT-D3 NMR spectra

To assign the NMR signals to the correct states, triple resonance backbone assignment experiments (CBCANH and CBCA(CO)NH) were first used to generate fragments of sequentially linked spin systems. Within each fragment, the two states could be distinguished by their differing Cα and Cβ chemical shifts. The boundaries of the fragments were formed by prolines, or residues with similar Cα and Cβ chemical shifts in the two states. To link the fragments, ^{15}N- and ^{13}C-NOESY-HSQC spectra were recorded, and NOEs between fragments identified. This allowed internally consistent state assignment for residues 256-288 and 294-313 to be assigned. For the remaining residues, no unambiguous inter-fragment NOEs could be identified, so the state could not be assigned. Note that correct state assignments are not required for calculation of the chemical shift difference between states.

Fitting of EXSY data

(^{1}H, ^{15}N) EXSY spectra (1) were recorded with mixing times between 0.1 s and 1 s, and intensities of well resolved peaks were fit using CCPN Analysis 2.3 (2). These intensities were used to calculate a parameter Ξ for each residue at each time point (3):

$$\Xi(t) = \frac{I_{AB}(t)\, I_{BA}(t)}{I_{AA}(t) I_{BB}(t) - I_{AB}(t) I_{BA}(t)} \cong k_{ex}^2 t^2$$

This treatment removes the effects of differential relaxation between the two states up to third order in time. The calculated Ξ values were fit to a quadratic model using R, and the confidence interval calculated using bootstrapping.

Supplementary tables

Supplementary table 1: Discarded hypotheses for the cause of the two-state behaviour of PACT-D3 observed in NMR spectra.

Hypothesis	Evidence
Monomer and dimer exchange	SEC-MALLS shows PACT-D3 is entirely dimeric at concentrations well below those used for NMR spectroscopy. NMR intensity and linewidth similar for both states.
Folded and misfolded exchange	TALOS-N secondary structure analysis and amide resonance dispersion indicates that both states are folded.
Bound ions from purification	The peak doubling is reproducible between samples, and remained in the presence of 10 mM EDTA.
Alternate disulphide bonding	Experiments were carried out in the presence of 10 mM TCEP, and side chain Cα and Cβ chemical shifts suggest that all cysteines are reduced.
Alternate histidine protonation states	A version of PACT-D3 with an H285A mutation showed two states by NMR, so protonation of H285 cannot be responsible for the two states. H300 is solvent exposed and distant from the residues with largest chemical shift differences between states, so is also unlikely to be responsible.
Proline isomerisation	Side chain Cβ and Cγ chemical shifts indicate that P280 adopts the cis isomer in both states.
Dimer domain swapping	Exchange between the two states is too fast – previous examples of domain-swapped homodimers exchange on a timescale of hours to days (4, 5)

Supplementary references

1. Farrow NA, Zhang O, Forman-Kay JD, Kay LE (1995) Comparison of the backbone dynamics of a folded and an unfolded SH3 domain existing in equilibrium in aqueous buffer. *Biochemistry* 34(3):868–78.
2. Vranken WF, et al. (2005) The CCPN data model for NMR spectroscopy: Development of a software pipeline. *Proteins Struct Funct Genet* 59(4):687–696.
3. Miloushev VZ, et al. (2008) Dynamic Properties of a Type II Cadherin Adhesive Domain: Implications for the Mechanism of Strand-Swapping of Classical Cadherins. *Structure* 16(8):1195–1205.
4. Ghasriani H, et al. (2014) Micelle-Catalyzed Domain Swapping in the GlpG Rhomboid Protease Cytoplasmic Domain. *Biochemistry* 53(37):5907–15.
5. Rousseau F, Schymkowitz JWH, Itzhaki LS (2003) The unfolding story of three-dimensional domain swapping. *Structure* 11(3):243–251.
6. Krissinel E, Henrick K (2007) Inference of Macromolecular Assemblies from Crystalline State. *J Mol Biol* 372(3):774–797.

Supplementary figures

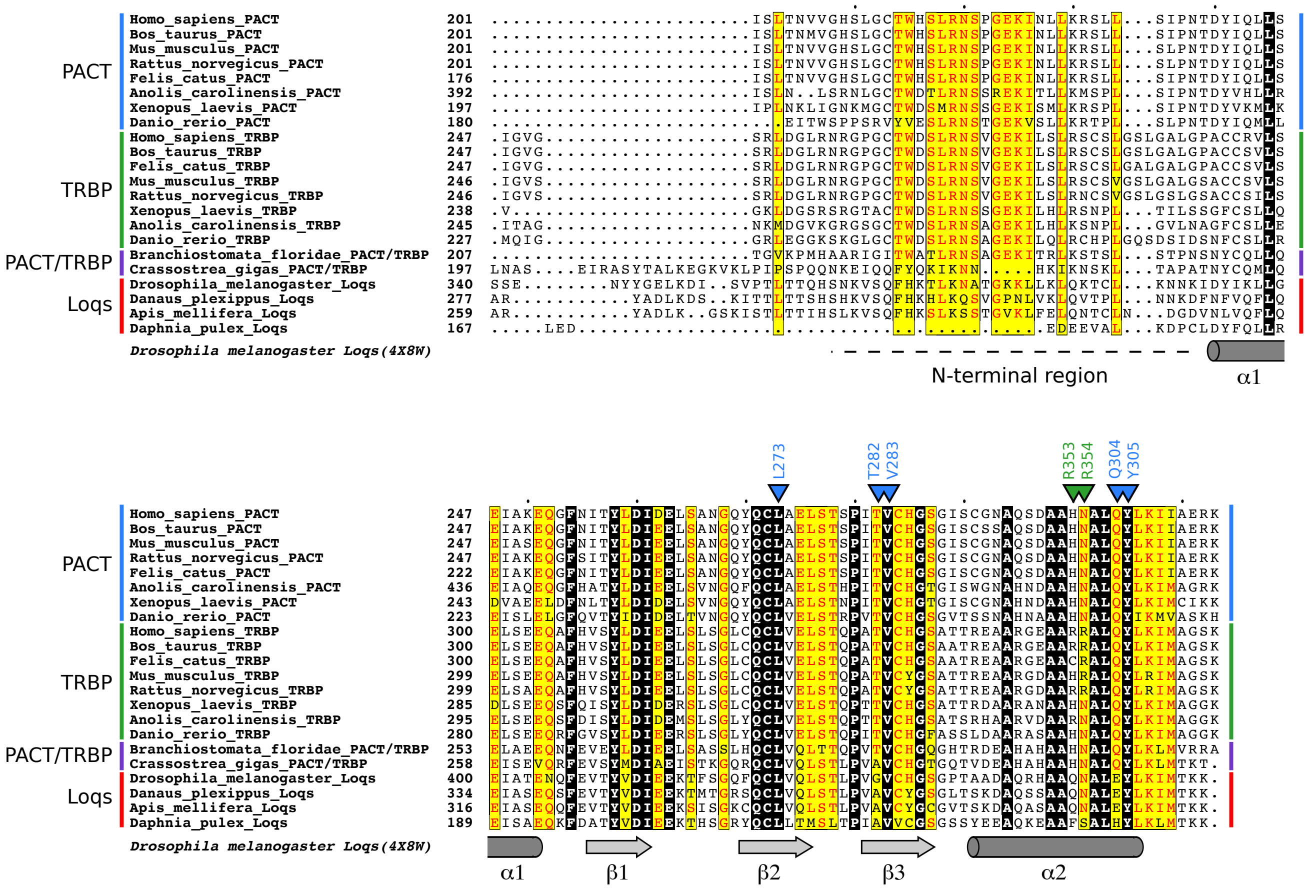

Figure S1

The amino acid sequences of PACT, TRBP and Loqs homologues were retrieved from NCBI, and aligned using ClustalX with default settings. The alignment of domain 3 is shown. The sequences are separated into groups on the left based on whether they are most similar to human PACT, TRBP or *Drosophila* Loqs. *Branchiostoma floridae* and *Crassostrea gigas* have only a single PACT homologue, which shares features with both PACT and TRBP. A yellow highlight with red text indicates >70% sequence identity; residues with 100% conservation are highlighted in black. Specific residues discussed in the main text are indicated above the alignment in blue (for PACT) or green (for TRBP). The secondary structure of Loqs D3 from PDB accession 4X8W is shown below the alignment.

UniProt accessions (in order shown in figure): O75569; NP_001039335; EDL27212; EDL79230; XP_006935445; XP_008116415; Q7ZYA5; XP_005172565; NP_599150; NP_001069146; XP_003988820; NP_033345; NP_001030113; XP_018100523; XP_008101858; NP_956291; XP_002587993; XP_011456094; AAF53295; EHJ63609; gnl|Amel_4.5|GB47214-PA; EFX79291. Note that the accession given for *Apis mellifera* Loqs is for the Amel_4.5_OGSv3.2_pep database of predicted protein sequences based on the *Apis mellifera* genome assembly 4.5, not for UniProt.

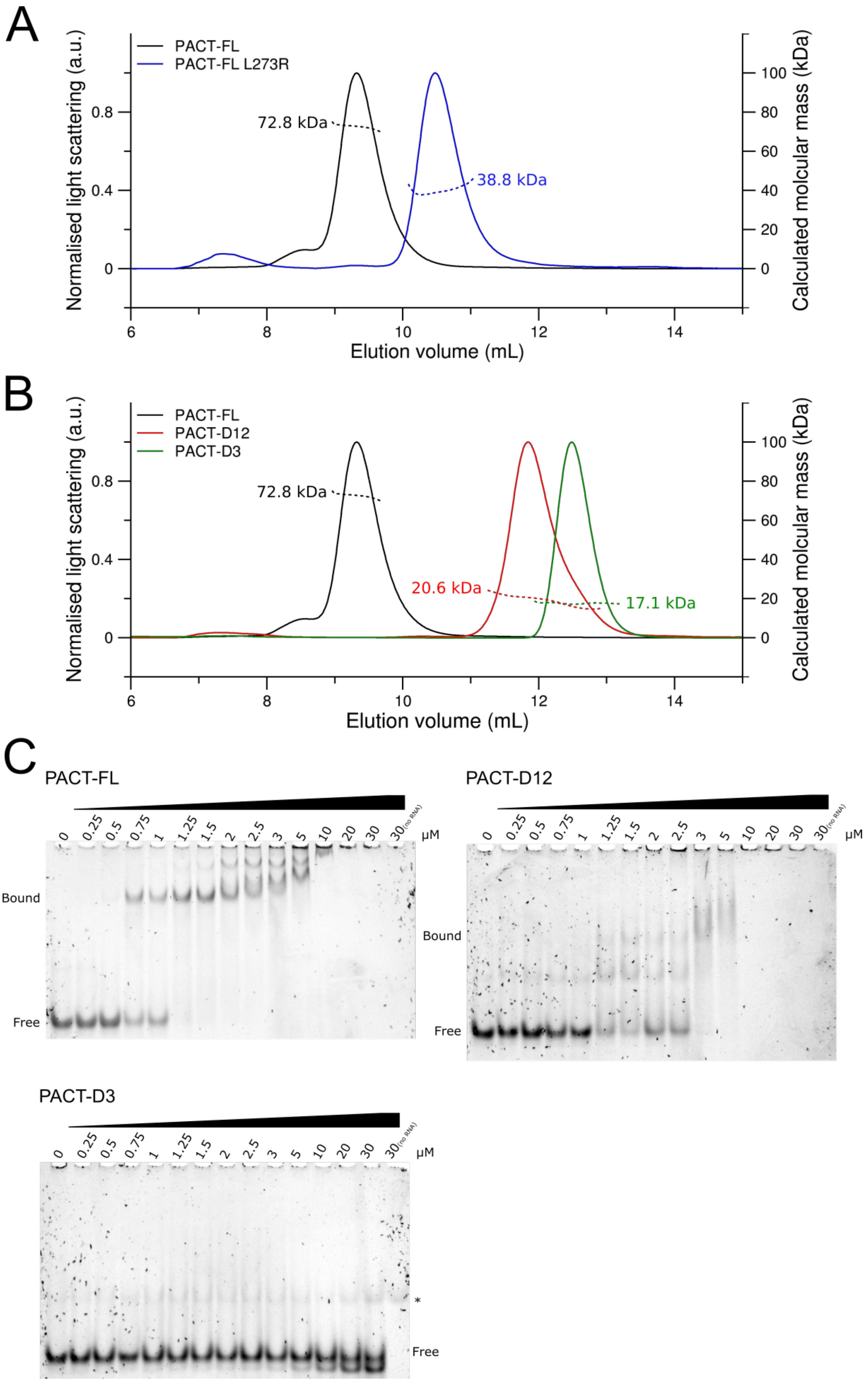

Figure S2

SEC-MALLS of A) FL PACT and FL PACT L273R; and B) FL PACT, PACT-D12 and PACT-D3. In these cases, the column was pre-equilibrated with 20 mM phosphate pH 6.5, 500 mM NaCl, 5 mM DTT and 5 % glycerol,

and data was processed using a dn/dc value of 0.165. C) Electrophoretic mobility shift assays testing for binding between PACT constructs and pre-miR-155.

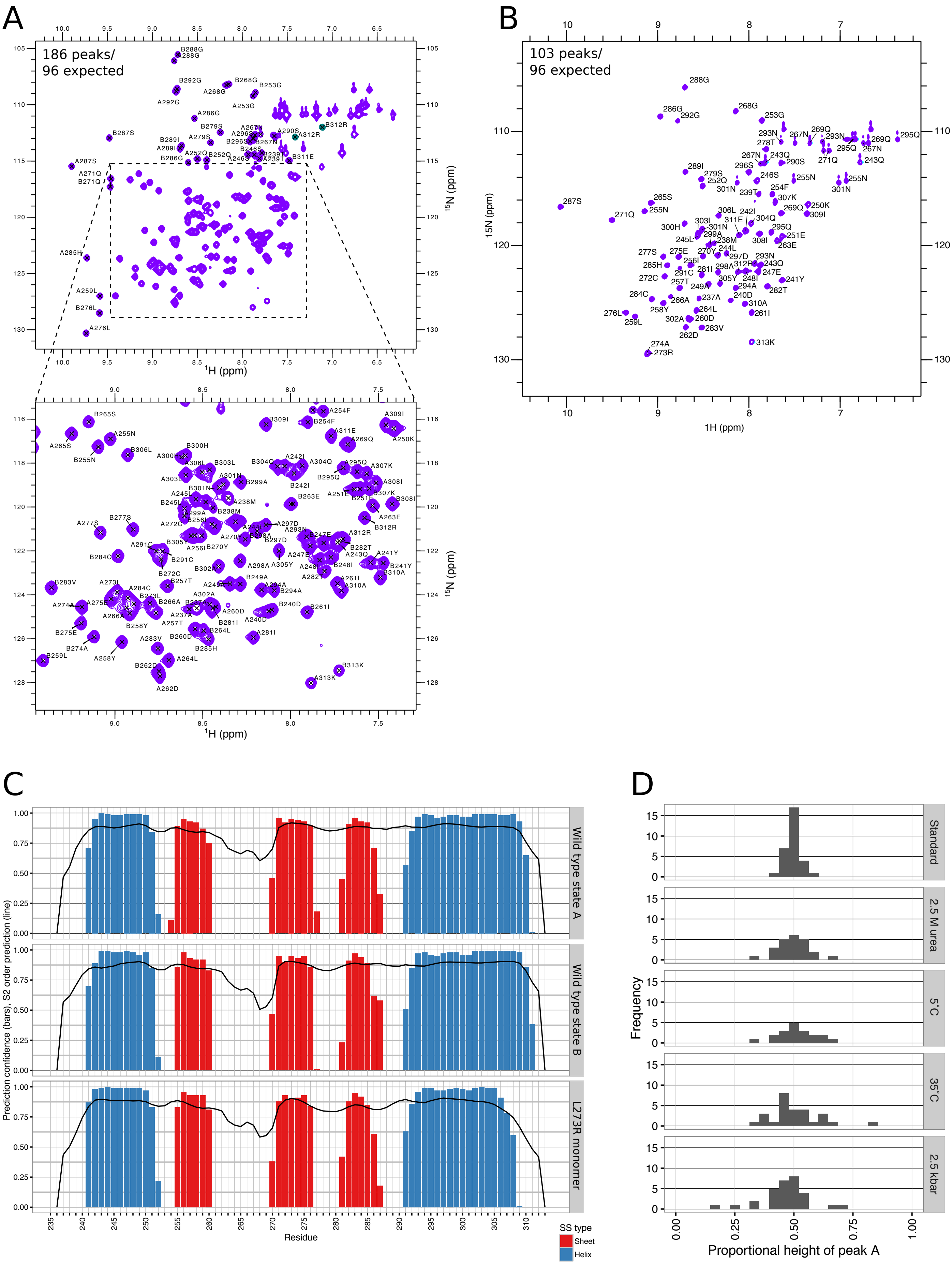

Figure S3

A) Assigned (^{1}H, ^{15}N) SOFAST-HMQC spectrum of WT PACT-D3. The observed and predicted numbers of peaks are shown in the top left. B) Assigned (^{1}H, ^{15}N) HSQC spectrum of PACT-D3 L273R. C) TALOS-N secondary structure predictions for PACT-D3 wt/L273R. The coloured bars show the confidence in the

secondary structure prediction, and the black line shows the predicted S^2 order parameter (lower values indicate greater flexibility). D) Histograms of the relative population of state A ($I_A/(I_A + I_B)$) for well resolved residues under different conditions. Regardless of temperature, pressure, or low concentration of denaturants, the distribution of populations remains centred around 0.5, showing that the populations of states A and B remain equal. Deviations from 0.5 are likely due to differential relaxation between the two states. Except for the condition being varied, all spectra were recorded at 25˚C at atmospheric pressure.

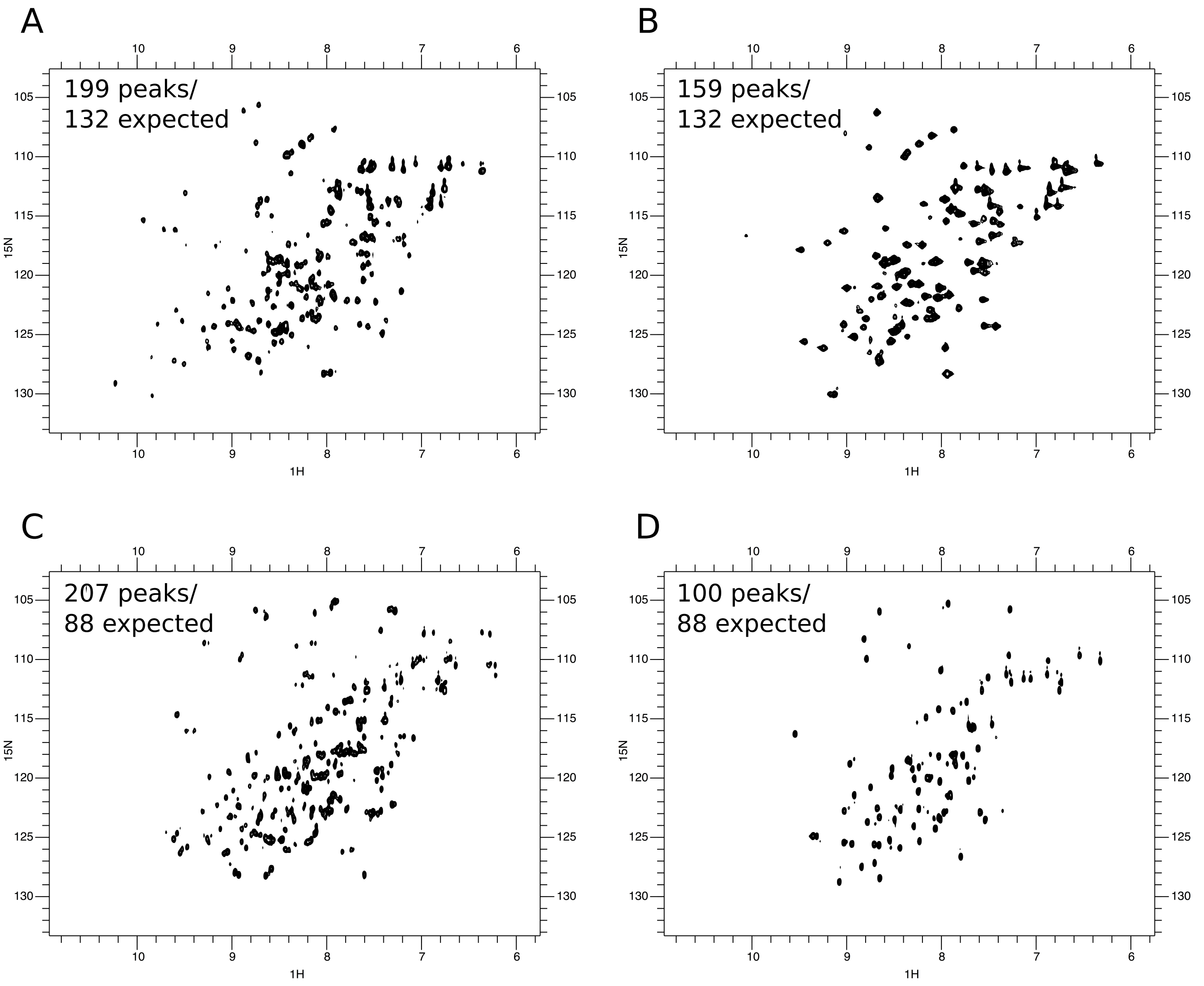

Figure S4

(^{1}H, ^{15}N) HSQC spectra of A) WT PACT-Ext-D3, B) PACT-Ext-D3 L273R, C) WT Loqs-D3, and D) Loqs-D3 L426R. The observed and predicted number of peaks is shown in the top left of each spectrum.

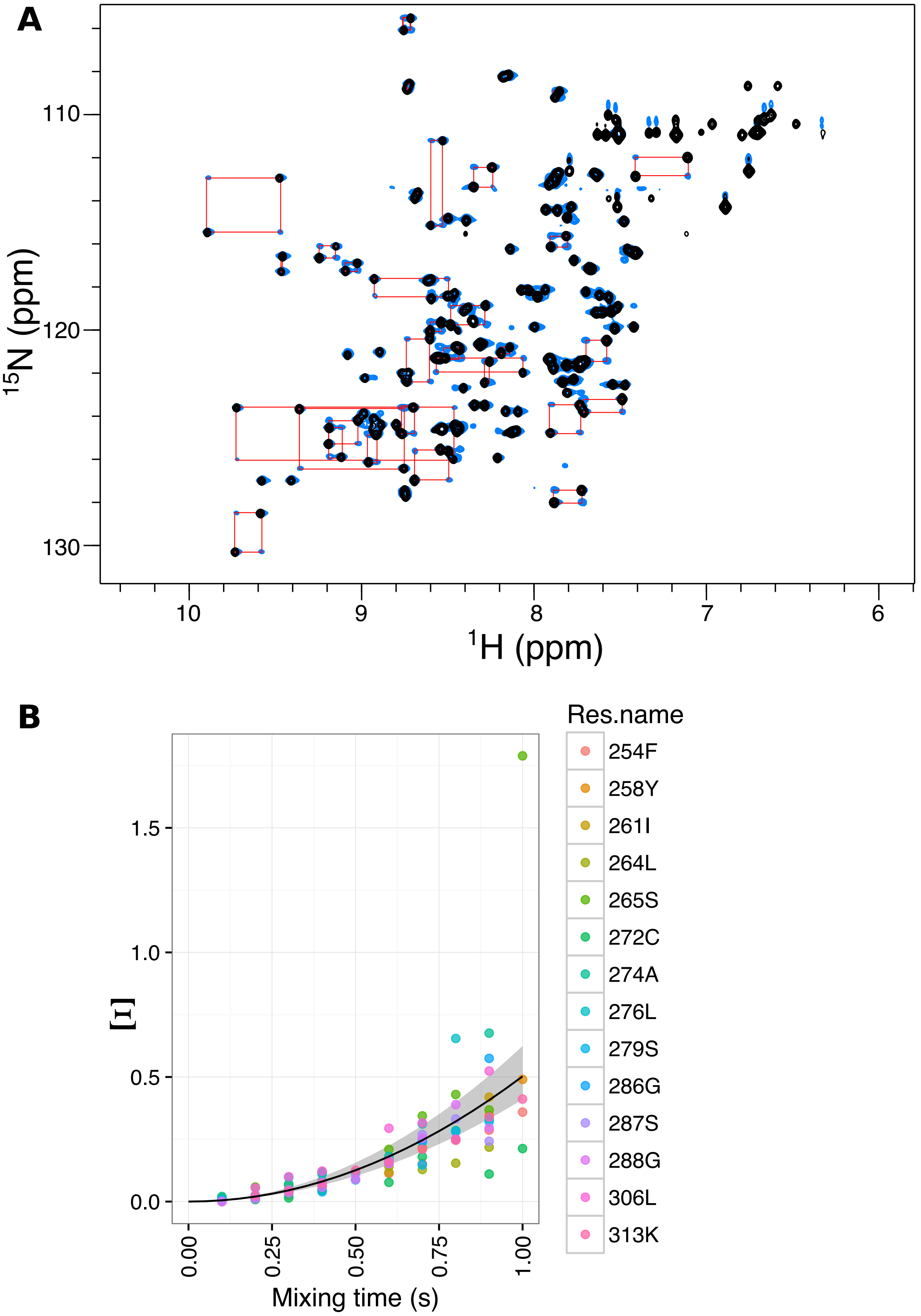

Figure S5

A) Overlay of a (^{1}H, ^{15}N) EXSY spectrum with a mixing time of 0.5 s (blue), and a (^{1}H, ^{15}N) SOFAST-HMQC (black) of wild type PACT-D3. Red boxes link auto and exchange peaks originating from a single residue. B) Plot of parameter Ξ against EXSY mixing time for each residue of wild-type PACT-D3 (see supplementary methods). The quadratic fit is shown as a black line, and the 95% confidence interval is displayed as a grey area. The fitted exchange rate was 0.71 s^{-1}, with a 95% confidence interval of 0.65 s^{-1} to 0.86 s^{-1}.

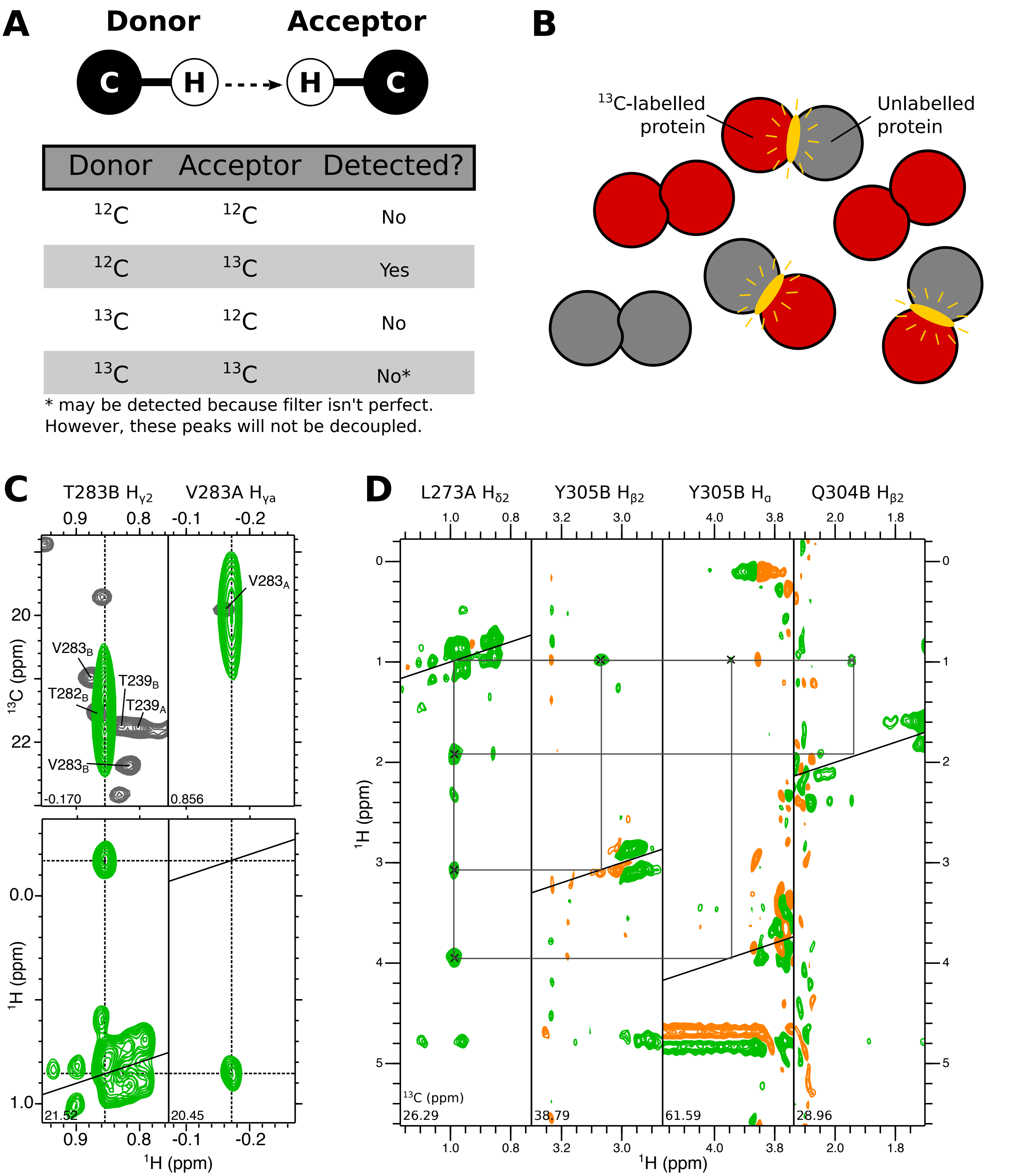

Figure S6

A) NOESY experiments detect magnetisation transfer between protons separated by less than about 5 Å. ^{13}C-filtered NOESY-HSQC experiments filter out some of these signals, leaving only cases where magnetisation was transferred from a proton attached to ^{12}C, to a proton attached to ^{13}C. In some cases, signals arising from protons attached ^{13}C are not fully suppressed. However, these cases can be recognised because no decoupling is applied during evolution of this proton, resulting in the signal appearing as a doublet. B) In a 50:50 mixture of ^{13}C-labelled and unlabelled PACT-D3, a mixture of ^{13}C/ ^{13}C-, ^{13}C/^{12}C- and ^{12}C/^{12}C-labelled homodimers will be present. The dimer interface of the ^{13}C /^{12}C-labelled homodimers is the only region in which ^{13}C and ^{12}C are in close proximity. Therefore, only intermolecular NOEs from the dimer interface are observed in ^{13}C-filtered NOESY-HSQC experiments. C) (^{1}H, ^{13}C) and (^{1}H,^{1}H) planes from the ^{13}C-filtered NOESY of PACT-D3, showing an intermolecular NOE between one of the γ methyl groups

from V283 in state A and the γ_2 methyl group of T282 in state B. The assigned (^{1}H, ^{13}C)-HSQC is shown in grey. The (^{1}H, ^{13}C) planes are shown to demonstrate that these NOEs can be assigned despite the large peak width in the ^{13}C dimension of the ^{13}C -filtered NOESY. D) Strips from a ^{13}C-filtered NOESY-HSQC of PACT-D3 showing intermolecular contacts between L273 $H_{\delta 2}$ in state A, and Q304 $H_{\beta 2}$ and Y305 H_{α} and $H_{\beta 2}$ in state B.

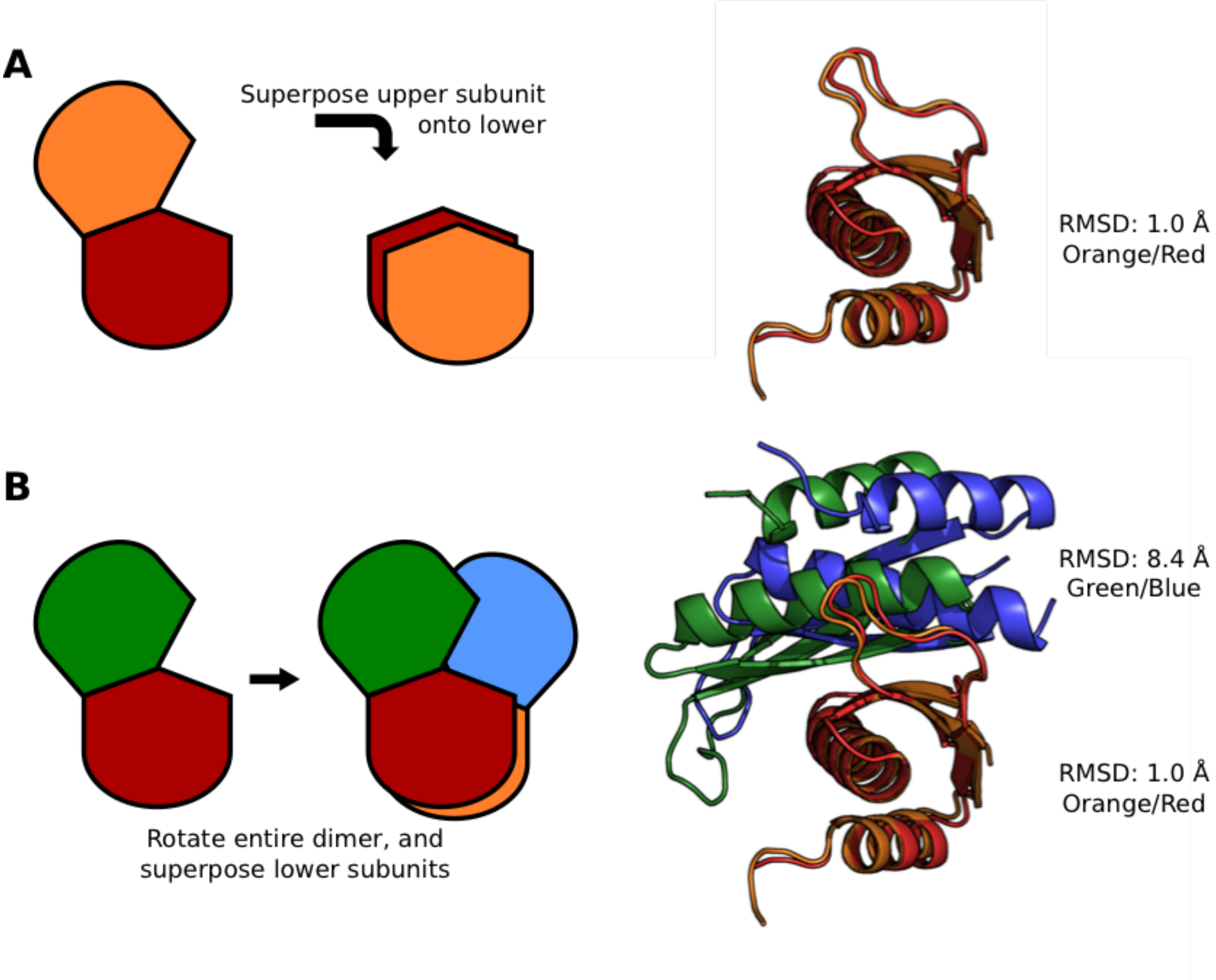

Figure S7:

Structural comparison of the two subunits of the Loqs-D3 dimer (chains A and D/F from PDB accession 4X8W). A) Superposition of the two subunits of the homodimer reveals only minor differences in structure between the two protomers, with a C_α RMSD of approximately 1 Å. B) However, if the entire homodimer is rotated and superposed so that two protomers (orange, red) are optimally aligned, the other subunits (blue, green) are displaced from one another by approximately 8 Å.

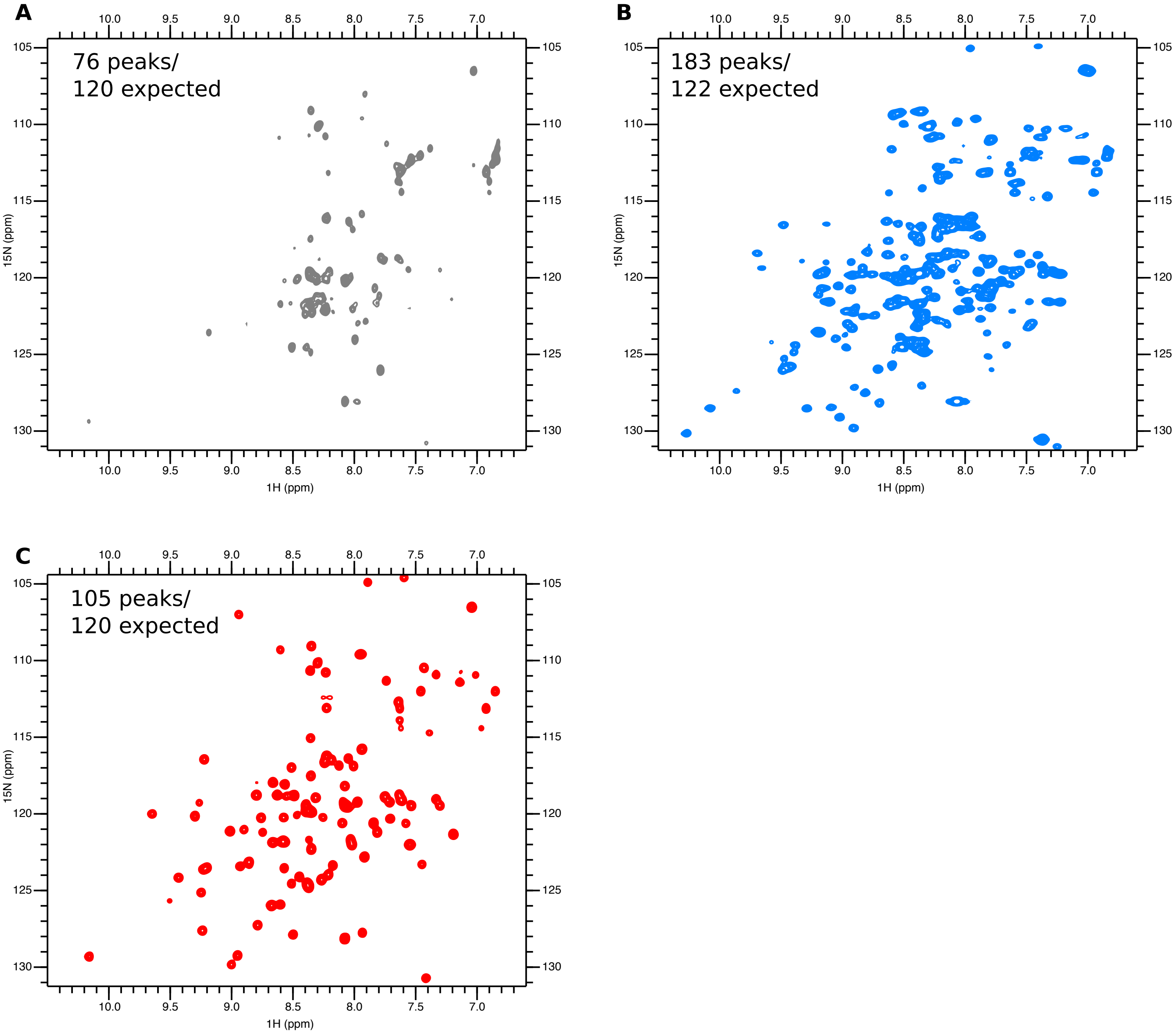

Figure S8:

A-C) (^{1}H,^{15}N)-HSQC spectra of A) wild-type TRBP-Ext-D3, B) TRBP-Ext-D3 R353H R354N, and C) TRBP-Ext-D3 L326R. The observed and predicted numbers of peaks are shown in the top left of each spectrum. The HSQC of TRBP-Ext-D3 shows fewer peaks than predicted, likely due to intermediate exchange between monomeric and asymmetric dimer states on the millisecond timescale.

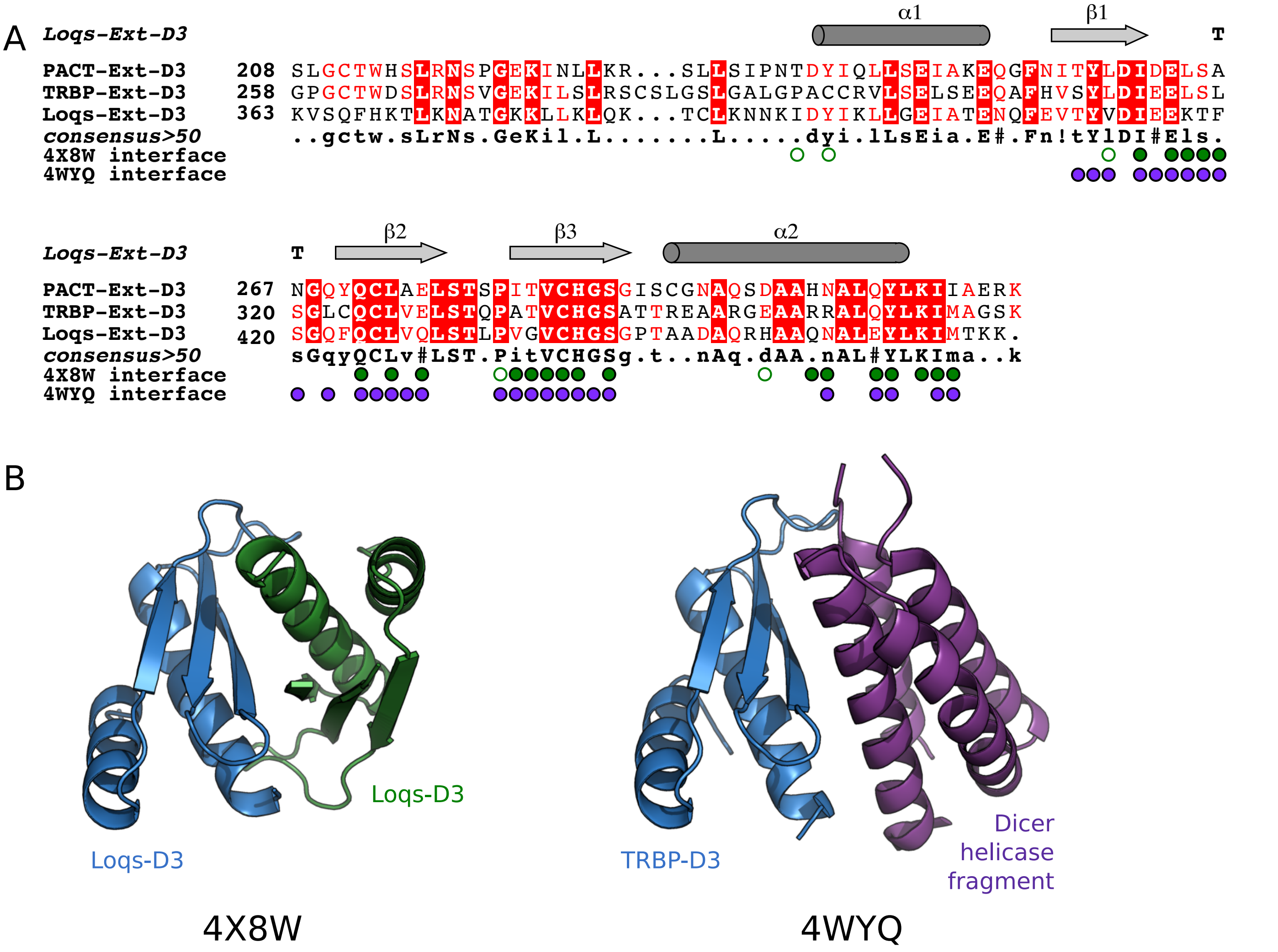

Figure S9:

A) Sequence alignment of PACT, TRBP and Loqs domain 3. The secondary structure of Loqs domain 3 (from 4X8W) is shown above, while circles below indicate residues at the Loqs homodimer interface (green) and the interface between TRBP and Dicer (purple). Open circles show residues that are only buried in one half of the Loqs-D3 homodimer. Surface residues were determined using PISA v1.51 (6). B) Comparison of Loqs-D3 dimer and TRBP-D3/Dicer-Ins structures.
